# Effective feature selection based HOBS pruned- ELM model for tomato plant leaf disease classification

**DOI:** 10.1371/journal.pone.0315031

**Published:** 2024-12-05

**Authors:** M. Amudha, K. Brindha

**Affiliations:** School of Computer Science Engineering and Information Systems, Vellore Institute of Technology, Vellore, Tamil Nādu, India; UPES Dehradun, INDIA

## Abstract

Tomato cultivation is expanding rapidly, but the tomato sector faces significant challenges from various sources, including environmental (abiotic stress) and biological (biotic stress or disease) threats, which adversely impact the crop’s growth, reproduction, and overall yield potential. The objective of this work is to build deep learning based lightweight convolutional neural network (CNN) architecture for the real-time classification of biotic stress in tomato plant leaves. This model proposes to address the drawbacks of conventional CNNs, which are resource-intensive and time-consuming, by using optimization methods that reduce processing complexity and enhance classification accuracy. Traditional plant disease classification methods predominantly utilize CNN based deep learning techniques, originally developed for fundamental image classification tasks. It relies on computationally intensive CNNs, hindering real-time application due to long training times. To address this, a lighter CNN framework is proposed to enhance with two key components. Firstly, an Elephant Herding Optimization (EHO) algorithm selects pertinent features for classification tasks. The classification module integrates a Hessian-based Optimal Brain Surgeon (HOBS) approach with a pruned Extreme Learning Machine (ELM), optimizing network parameters while reducing computational complexity. The proposed pruned model gives an accuracy of 95.73%, Cohen’s kappa of 0.81%, training time of 2.35sec on Plant Village dataset, comprising 8,000 leaf images across 10 distinct classes of tomato plant, which demonstrates that this framework effectively reduces the model’s size of 9.2Mb and parameters by reducing irrelevant connections in the classification layer. The proposed classifier performance was compared to existing deep learning models, the experimental results show that the pruned DenseNet achieves an accuracy of 86.64% with a model size of 10.6 MB, while GhostNet reaches an accuracy of 92.15% at 10.9 MB. CACPNET demonstrates an accuracy of 92.4% with a model size of 18.0 MB. In contrast, the proposed approach significantly outperforms these models in terms of accuracy and processing time.

## 1. Introduction

Agriculture is a crucial sector that plays a vital role in supplying important resources such as food and raw materials for human consumption and numerous businesses. However, it faces a significant obstacle in the form of plant diseases, which have a worldwide impact. Globally, tomatoes (Solanum lycopersicum) are one of the most important vegetable crops, with China leading production at 68.2 million tons, followed by India at 20.7 million tons, Turkey at 13 million tons, and the United States at 10.2 million tons. Countries that are in the European Union are among the other important producers. India holds the second place both in tomato production and in tomato farming [1]. Diseases severely impact tomato crops, resulting in substantial losses for the agricultural sector. For example, early blight is one of the most widespread diseases worldwide and can result in large fruit lesions and productivity losses. Similar to late blight, late blight can be extremely damaging in regions with humid climates and seriously harm crops. Improving the quantity and quality of the crops depends on protecting tomatoes from illnesses. In order to select the best course of action and prevent the disease from spreading, it is crucial to provide early disease detection and identification. These diseases possess the capacity to cause significant harm to crops, leading to reduced yields, financial difficulties for farmers, and concerns over food security [2]. Tomato plants are vulnerable to several infections, such as bacteria, fungi, viruses, nematodes, and other microbes. These detrimental substances might potentially target several parts of the tomato plant, including the leaves, stems, roots, and fruits, resulting in a wide range of symptoms. Common indications of the condition may manifest as leaf spots, wilting, discoloration, fruit rot, or stunted development [3]. The diseases exhibit a wide range of symptoms, which may include aberrant fruiting, leaf spottiness, stunted growth, withering, or discoloration, depending on the particular disease and the plant species that is impacted.

Over the past few years, the integration of artificial intelligence (AI) technologies has presented promising prospects for the automated classification of diseases affecting tomato plants. When it comes to advanced methods, deep learning (DL) models, which include artificial neural networks (ANN), convolutional neural networks (CNN), and transfer learning (TL)-based network architectures, have shown great success in identifying plant diseases [4]. The main goal of this study is to increase the degree of accuracy, improve computational efficiency, and expedite the rate of convergence of DL models used to classify diseases damaging tomato plants. In recent years, CNN research has made substantial progress because to advancements in parameter optimization, the use of regularization techniques and improvements to activation functions and accompanying loss functions. Throughout history, neural network training has been a laborious and time-intensive process, frequently requiring several days or even weeks to finalize. This long duration posed a significant challenge, particularly in research conducted in real-time environments whereby quick computation is vital [5]. Therefore, there is a crucial need for enhanced computational speed to meet the requirements of current popular applications. Techniques such as pruning and compression have evolved as effective strategies to address this difficulty. Pruning a deep learning network is essential for improving memory and energy efficiency, resulting in faster training and improved accuracy [6]. There has been a considerable amount of progress made in this field throughout the span of the past few decades, due to the adoption of an extensive number of innovative pruning procedures. These techniques can be classified into a number of different categories, including layer-wise pruning, structured pruning, pruning weight, pruning neuron, and pruning filter. Within the scope of this paper, a systematic weight pruning strategy is presented with the intent of locating and removing the weights from neural networks that are considered the least important while maintaining the performance of the model [7].

The cutting-edge deep convolutional Extreme Learning Machine (DC-ELM) architecture outperforms previous deep learning architectures in terms of accuracy. However, training DC-ELM still faces significant computational costs. This method requires a significant amount of space and time [8]. The deep learning techniques with several architectures including CNN, pre-trained models, long short-term memory and capsule neural network have widely been employed in the application of the image data. In case of tomato plant leave diseases, used deep learning model for classification have several limitations: Lack of robustness in managing differences in leaf textures, shapes, and lighting conditions, resulting in lower accuracy in real-world circumstances. Early disease detection requires high-quality, well-labeled datasets, which may not always be available. Each plant species and disease requires a separate model due to limited generality. Complex CNN architectures have low computational efficiency and high memory requirements, rendering them unsuitable for implementation on resource-constrained systems. Interpreting model conclusions and understanding illness detection aspects is difficult, reducing model transparency and dependability.

The main goal of this research is to minimize the size of the model by using a connection pruning strategy to maximize computing performance. Previously developed pruning methods focused on evaluating the importance of each neuron and eliminating those that were redundant. As the number of hidden layer neurons increases, over fitting can occur, reducing the model’s ability to generalize. By removing the least significant connections, this approach creates a faster and more efficient model. In this research, a hybrid CNN-ELM model is proposed, in which CNN uses an optimized dataset to extract significant features while ELM acts as a lightweight classification technique. The Hessian-based Optimal Brain Surgeon (HOBS) Pruning algorithm optimizes the neural network by eliminating unnecessary connections, hence improving computing efficiency while maintaining accuracy. This framework, commonly known as inventive architecture, tackles both accuracy and computational speed. Pruned connections show minimal impact on the overall loss function, highlighting significant superfluity or redundancy. The significant contributions of this work outlined as follow:

- The dataset undergoes pre-processing, including resizing and applying a Gaussian filter to enhance image quality and feature extraction.
- The Elephant Herding Optimization (EHO), a meta-heuristic technique suggested to improve classification by selecting the most relevant features, is used to accomplish optimal feature selection.
- A pruned Convolutional Neural Network (CNN) combined with HOBS-ELM is employed as the classifier network to effectively classify diseases in tomato crops.
- The model’s performance is evaluated on a benchmark dataset and compared with existing approaches, demonstrating its superior effectiveness.

The paper’s organization consists of the following sections: Section II examines previous research in the field, outlining its strengths and weaknesses as well as the suggested study. In Section III, the preliminary research is presented together with a description of the suggested approach. Section IV investigates the chosen dataset, presenting the findings and discussion. Section V concludes by discussing the study’s findings and making recommendations for future research.

## 2. Review of related work

Currently, the focus of research in the domain of tomato leaf disease categorization is predominantly on the advancement of novel approaches that apply neural network architectures. The objective of these activities is to optimize and decompose neural networks in order to improve computational efficiency, specifically for embedded systems, and facilitate the classification of diseases in real-time. These developments can be used to integrate intelligent systems that reduce crop production losses, remove labor-intensive manual monitoring, and reduce human interaction [9]. A multi-step methodology has been implemented by researchers in order to optimize the performance of disease classification models. The process begins with the application of principal component analysis to reduce the size of image datasets, followed by the incorporation of the whale optimization algorithm to extract the most essential features [10]. The suggested location feedback classification network is a novel self-supervised model. Without the need for human annotations like bounding boxes or certain segments, this algorithm can autonomously identify informative areas in onion images. Such informative regions are located by the location network and then assessed by the feedback network. Next, these traits are used by the classification network to accurately classify tomato plants as either healthy or diseased [11]. The author has created a hybrid Convolutional Neural Network (CNN) model specifically built to classify diseases in tomato plants. This model combines Support Vector Machines (SVM) and an attention module to enhance the feature extraction and classification procedures. This model has undergone rigorous testing on a comprehensive dataset of tomato leaf images, and the results demonstrate an exceptional level of accuracy [12].

The author of [13] used a convolutional neural network (CNN)-based approach that accurately identifies the locations on tomato leaves that are affected by diseases. This methodology improves the accuracy of disease identification and provides valuable insights into the appearance of symptoms and the localization of the disease. Even though the training takes longer and the computing complexity is higher. In the context of [14] introduces an AI-driven framework designed for the detection and classification of prevalent guava plant diseases. The framework utilizes 4E color difference image segmentation to isolate regions affected by the disease. Additionally, it employs color (RGB, HSV) histogram and textural (LBP) features to extract comprehensive and informative feature vectors. The author of the study in [15] fruit classification and quality recognition research in computer vision, machine learning, and pattern recognition. As the best classifier, ResNet-18 had 99.8% accuracy. The paper acknowledges the dataset’s limitations, such as its limited size, and offers deep learning enhancements for fruit quality classification. In contrast, in the study described in [16], a unique strategy integrating dense and deep residual networks gives Restructured deep residual dense network a hybrid deep learning model for accurately identifying tomato plant leaf diseases. This model combines the best features from both architectures. This model effectively decreases the number of training parameters, resulting in an average identification accuracy of 95%, indicating a promising potential for enhanced disease detection in tomato plants.

To tackle issues with imbalanced datasets and crucial feature extraction, the Multilevel Feature Fusion Network (MFFN) was created, as mentioned in [17]. However, it has a limited capacity to differentiate between diseases that have similar symptoms. Despite the presence of complex attention mechanisms within MFFN, misclassifications may still occur in cases where diseases are highly similar to one another. To classify tomato leaf diseases, the author presented a unique architecture in [18] called the convolutional block attention module. These lightweight models were developed, and it is significant that the attention-enhanced model yielded better detection accuracy even though it had a slight rise in network complexity and parametric quantity compared to the version without attention blocks. To improve CNN accuracy, the author presented a new idea in [19] that makes use of fuzzy logic. This enhancement was achieved by swapping out the conventional pooling layer in the thinNet architecture that was developed for lemon fruit classification. One major issue with this method is that it uses back propagation during training, which limits its computing efficiency. The repeated optimization characteristic of back propagation makes it computationally expensive and time-consuming, which limits its scalability, especially in situations with bigger datasets and environments with limited resources.

This study introduces [20] an improved ResNet-50 deep learning classifier that is capable of detecting tomato leaf diseases with an accuracy of over 95%. The model exhibits exceptional performance in a variety of environments by employing sophisticated data augmentation and transfer learning. In the study described in [21], YOLOv4 for pepper target detection, by adding Mosaic data augmentation and Convolutional Block Attention Module to the network, the technique improves learning, reduces interference, and prioritizes significant information.

To identify diseased tomato plants, the authors of [22] presented a hybrid CNN model that relies on deep segmentation. To improve object detection, this hybrid model uses instance semantic segmentation approaches with pre-trained UNet and SegNet. While the model performs admirably for more extensive pixel ranges, it suffers when confronted with narrower ones. When it comes to semantic segmentation, one area where this approach falls lacking is when lesions overlap and the model fails to classify the overlapping areas. A further limitation introduced by this hybrid model is the relative shortage of image datasets. The author in [23] presented a pruning strategy that involves the direct removal of insignificant filters, without compromising test accuracy. This iterative procedure generates simpler models while maintaining comparable accuracy. Significantly, this approach effectively addresses the obstacle of eliminating interconnected feature maps, a limitation in earlier techniques for removing filters in CNNs. The approach proposed in reference [24] ignores the necessity for costly cycles of pruning and fine-tuning by conducting a singular pruning operation on pre-trained architectures. The substantial computational demands associated with training the model from its inception, however, render this approach inaccurate. In reference [25], the author introduced CACPNET, a lightweight model inspired by ResNet that makes use of channel attention and channel pruning. By employing L1-norm channel weights and local compression ratios, it eliminates irrelevant channels, thereby optimizing the conditions for feature extraction. Complexity and model parameters are thereby reduced. The study examined [26] the graph-based Interactive Image Segmentation creates the background uniformity across experimental and real-world datasets, improving model adaptability. Optimal for agricultural platforms with limited resources, channel pruning decreases model size and computing effort by 85.19% and 92.15%, respectively, while maintaining accuracy.

These difficulties include the utilization of large model sizes, excessive memory consumption, and significant computational requirements. The existing approaches utilized for tomato plant disease classification and detection are summarized in Table 1. Model pruning is a feasible approach that allows for the selective targeting of particular levels such as weight, filter, channel, or layer, thereby efficiently addressing these difficulties. In order to overcome these limitations, an innovative approach for classifying tomato plant diseases using pruned ELM is proposed.

**Table 1:** Characteristic and limitations of existing work.

References	Method	Crop Name	Characteristic	Limitations/Gap	Accuracy%
[27]	SVM, Naive Bayes, Decision tree, K-nearest neighbor (K-NN), Random Forest, Logistic Regression and Decision Tree	Tomato	The model employs image segmentation and Histogram of Oriented Gradients (HOG) for feature extraction, training various machine learning classifiers.	HOG may not capture all relevant information.	92 57 65 68 79 87
[28]	CNN Network	Tomato	A CNN-based model has been developed to detect diseases in tomato crops. The findings demonstrate that this model outperforms pre-trained models such as VGG16, InceptionV3, and MobileNet.	It takes more epochs to train the model.	91.20
[29]	MobileNet v2-YOLOv3	Tomato	MobileNetv2-YOLOv3 strikes a balance between accuracy and real-time detection by introducing the GIoU bounding box regression loss function and employing a lightweight mobile network model.	Only the disease tomato gray leaf spot was taken.	92.53
[30]	Semantic segmentation neural network	Tomato	Unlike existing networks, the proposed neural network reduces image resolution loss during feature extraction.	Adding new classes requires additional parameters.	80.2
[31]	Mask RCNN-ResNet101	Tomato	This novel study introduced deep CNN models using U-Net and Mask RCNN architectures for exact segmentation.	The dataset size is insufficient.	82.72
[32]	ResNet50, ResNet18, and ResNet101	Tomato	The ResNet101 model, trained with the augmented dataset, performs better with higher accuracy.	It takes longer training time to train the model.	95.83
[33]	CapsNet	Tomato	CapsNet effectively depicts spatial relationships within images and demonstrates a high level of accuracy in classifying tomato leaf diseases. It provides a promising alternative to conventional CNNs.	Feature selection technique is not performed.	96.39
[34]	CapsNet, MobileNetV2	Tomato	MobileNetV2 architecture, SiLU6 activation function, and capsule networks form a hybrid framework. This approach estimates plant disease severity. Features were extracted using multilateral MobileNetV2 networks.	The dynamic routing method makes CapsNet more computationally intensive than CNNs.	94.47
[35]	CNN-Recurrent neural network (RNN)	Tomato	The robust model produces accurate and superior results.	Complex architecture, more training time, increased computational resources.	81.75
[36]	EfficientNetv2 and Swin transformer	Tomato	The proposed model trained on balanced dataset.	Longer training time, numerous parameters.	99.70

The goal is to address issues related to preprocessing complexity, model size, and classifier performance. This method addresses unbalanced data and similarities between class features. In order to optimize the design of the model, techniques for feature extraction, selection, and pruning are integrated, drawing upon insights from prior research on the classification of plant diseases. Additionally, CNN methods are used to improve disease categorization in tomato plants.

## 3. Preliminaries and proposed methodology

This research employs a quantitative, descriptive, exploratory, and correlational approach to investigate various methods for identifying plant diseases, emphasizing machine learning and deep learning techniques. Traditional approaches often omit deep learning, but our study incorporates both machine learning and advanced deep learning models. The classification framework categorizes tomato plant leaves based on health and specific diseases caused by various pathogens: viral infections such as tomato yellow leaf curl, bacterial infections like bacterial spot, and fungal infections, including early blight, late blight, leaf mold, septoria leaf spot, and target spot. Damage from spider mites is also addressed. The Elephant Herding Optimization (EHO) algorithm [37], the pruned ELM classifier, and Pearson’s correlation analysis are all significant contributions to this study. EHO, a swarm-based metaheuristic inspired by the cooperative behaviors of elephant herds, identifies optimal feature subsets. By simulating these herding dynamics, EHO directs the search process to select the most informative features from a dataset of tomato leaf images, enhancing model performance for disease classification.

Pearson’s correlation coefficient is utilized to analyze the linear correlations among selected features, aiding in the identification of features with significant predictive value and low redundancy. In the subsequent phase, the pruned-ELM [38] deep learning model is employed to automate disease classification with enhanced computing efficiency and precision. This integration of EHO, Pearson’s correlation, and pruned-ELM represents a significant contribution, leading to improved accuracy and efficient memory utilization.

### 3.1 Dataset description

**Fig 1:**
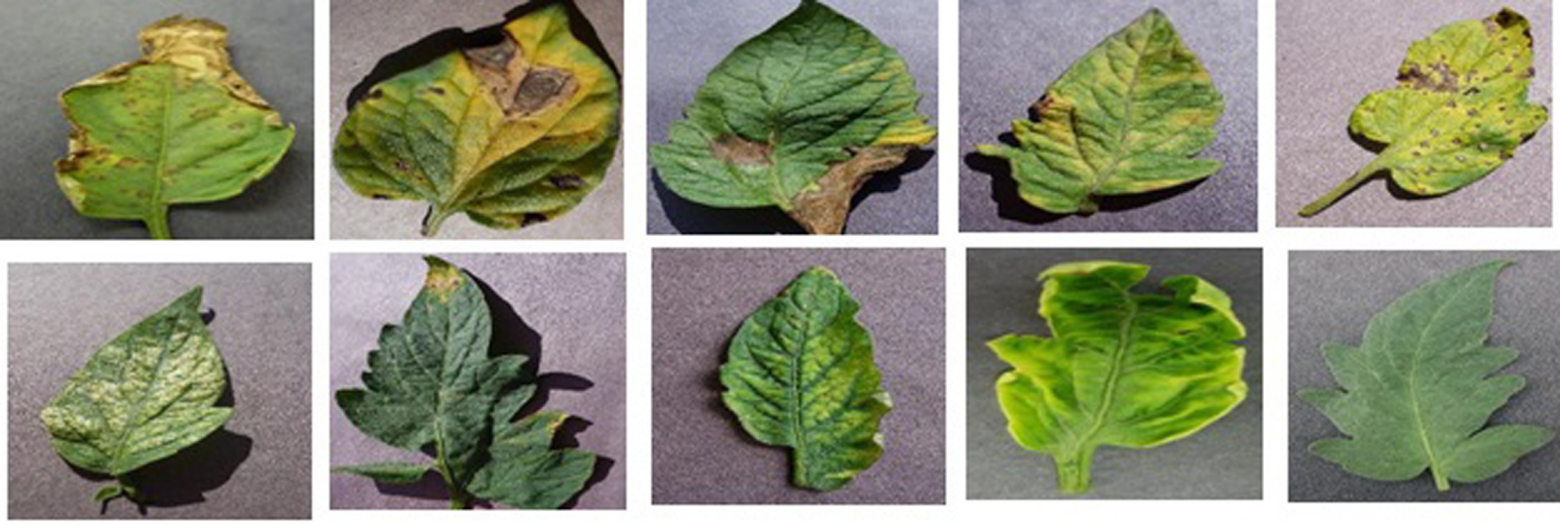
Sample images of tomato leaf disease dataset. (a) Bacterial spot, (b) Early blight, (c) Late blight, (d) Leaf Mold, (e) Septoria Leaf spot, (f) Spider mites, (g) Target Spot, (h) Mosaic virus, (i) Yellow Curl Virus, (j) Healthy.

The model’s performance was evaluated using tomato plant leaves images dataset was obtained from the open-access repository that can be found at www.PlantVillage.org. It contains a collection of more than 50,000 images of leaves. Images of tomato leaves were specifically extracted for the purposes of our research. An overview of our dataset is given in Fig 1, which provides a summary of the data. Our dataset contains eight thousand images, which are organized into nine different diseases and healthy images [39]. To ensure fair model performance across all categories and avoid potential performance biases due to imbalanced sample distribution, data augmentation techniques were applied. These techniques were specifically used on images showing both healthy and diseased leaves. The dataset is extensive and covers both single disease and multiple disease instances on tomato leaves, primarily caused by biotic stress factors [40]. To facilitate training and testing, the dataset was segregated into separate subsets, maintaining a ratio of 20% for testing data and 80% for training data. The training set of the dataset consists of 8000 images, while the test set contains 2000 images.

This dataset covers a wide range of tomato plant conditions, encompassing both healthy and diseased states, with annotations provided by field experts [41]. This study’s major goal is to assess and classify tomato plant leaves according to their health, with a focus on differentiating between diseases like yellow leaf curl virus (TYLCV), bacterial spot (Xanthomonas campestris), early blight (Alternaria solani), late blight (Phytophthora infestans), leaf mold (Passalora fulva), septoria leaf spot (Septoria lycopersici), spider mites (Tetranychus urticae), and target spot (Corynespora cassiicola), mosaic virus (ToMV) in addition to a category indicating ’healthy’ plants.

### 3.2 Data preprocessing

Pre-processing is an essential step in improving the quality of input images, specifically in the context of classifying plant diseases. Plant sample images are gathered in the early stages and subsequently transferred to the pre-processing phase. In this instance, a Gaussian filter is applied to the image to effectively eliminate noise, resulting in more distinct and informative images of the plant leaves. Furthermore, to ensure consistency in input dimensions and optimize compatibility with subsequent processing stages in our classification model, the image size is standardized to 224×224. This method combines deep learning techniques for preliminary feature extraction and optimizes the selection process through an EHO-inspired optimization mechanism.

The analysis was conducted using TensorFlow, Keras, and scikit-learn. TensorFlow and Keras were utilized for model development, while scikit-learn assisted with feature evaluation and optimization.

### 3.3 Convolutional neural network

**Fig 2:**
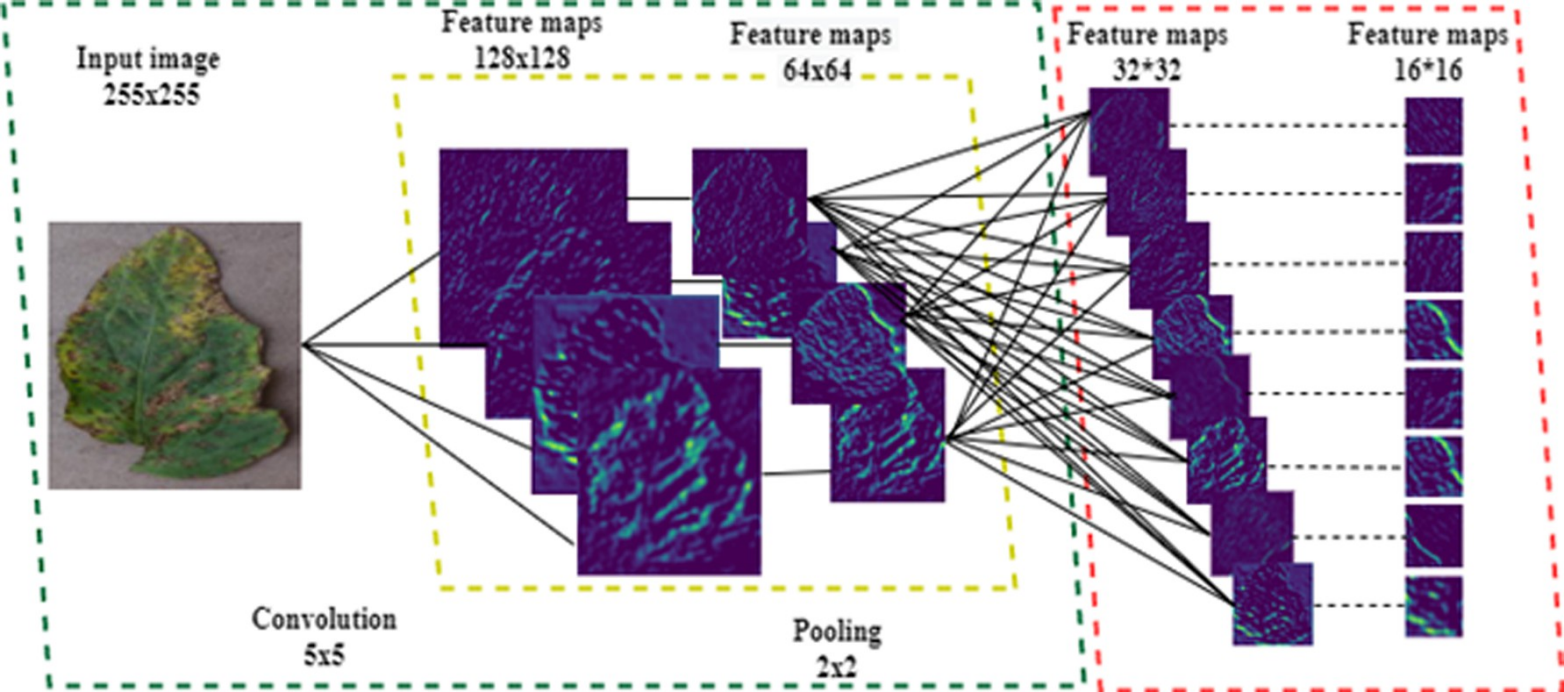
Input image and extracted features maps.

To effectively evaluate susceptibility to diseases in various regions of tomato leaves is essential to discern unique characteristics within these leaf sections. Improving the model’s efficiency relies on extracting pertinent features from leaf images, is illustrated in Fig 2. This process entails feeding the images into convolutional neural network and choosing specific intermediate layers to extract features. These extracted features are later flattened to create a feature vector for each image, enabling thorough analysis.

Utilizing convolutional neural networks (CNNs) proves to be a potent technique for feature extraction. The CNN architecture predominantly comprises initial convolutional layers combined with pooling layers, working together to acquire significant features from the input target images. Selected features are subsequently derived from chosen intermediate layers, resulting in the creation of a feature vector by flattening the extracted features. The CNN architecture for the extraction of features from tomato leaf images is devised as follows Inception begins with an initial convolutional layer, comprising 16 filters of dimensions 5x5, generating 16 feature maps, each with dimensions 128x128. Following this, a pooling layer employs a 2x2 window to generate 16 characteristic maps, each measuring 64x64. Subsequent to this stage, another convolutional layer, armed with 32 filters, processes these maps, followed by a pooling layer, which yields feature maps of dimensions 32x32x32. The progression continues with an additional convolutional layer featuring 64 filters, further processing these maps, leading to a pooling layer that generates feature maps measuring 16x16x64. Final convolutional layer with feature maps measuring 16x16x64 will generate feature vectors with the following number of components. The spatial dimensions of this layer are 224 x 224, and it has three channels (RGB). As a result, the final convolutional layer will extract 16,384 features. The flattening layer will flatten these features into a one-dimensional array before passing them along to the fully connected layers for further processing. To abstract the features further, fully interconnected layers are used, and the flattening layer eventually generates the ultimate output label probabilities by applying a softmax activation function. The extracted features are then passed to the classification layer for the purpose of classification.

### 3.4 Elephant herding optimization

This work describes the Elephant Herding Optimization (EHO) algorithm [42], a metaheuristic swarm-based approach to feature selection that improves the accuracy of classifying tomato plant leaf diseases. The EHO algorithm is employed following the initial step of training, which involves feature extraction using Convolutional Neural Networks. The suggested EHO-based feature selection method uses optimization concepts to identify features that minimize classification time while increasing accuracy. This novel approach highlights relevant features unique to datasets on tomato plant leaf diseases, making classification tasks easier. EHO uses cooperative behavior observed in elephant herds to select important features while continuously optimizing the feature space of tomato plant leaf datasets. The chosen features improve the accuracy and efficiency of disease classification and visual inspection of the leaves of tomato plants.

This algorithm derives its inspiration from the social structure observed within elephant groups. Elephants, in their natural habitat, exhibit complex social behaviors, with distinct roles for both female and male elephants. The foundation of elephant social organization rests on the formation of various clans, with the matriarch, the eldest female elephant, assuming the role of clan leader. Each clan comprises one female elephant along with other family members. Typically, female elephants maintain group cohesion, but as male elephants mature, they tend to depart from their family groups. The simulation of EHO incorporates operations akin to clan updating and clan separating, mirroring the real-world dynamics of elephant herding behavior [43]. In the process of selecting the feature, the goal is to pinpoint the optimal subset of features that enhance classification analysis outcomes. Within elephant groups, various clans emerge, each led by a matriarch and maintaining a consistent number of elephants. In every generation, a distinct group of male elephants resides apart from their herds, representing the minimum fitness value in maximization problems. In contrast, the matriarchs embody the maximum fitness value [44].

#### 3.4.1 Fittest elephant’s position update using clan influence

The positions of elephants within a clan are adjusted according to both the matriarch (the fittest elephant) and the elephant positioned at the centre of the clan. The matriarch’s position (the fittest elephant) is updated in relation to the elephant at the central position of the clan.


$$E_{next}=E+K*\left(E_{bestC_{I}}-E\right)+m*\left(E_{globalC_{i}}-E\right)$$ (1)

In this Eq 1, *E* is the current solution for the elephant, "*E*_*next*_" is the next solution for the current elephant, the above equation combines the influences of the local best feature subset $\left("E_{bestC_{I}}"\right)$ and the best subset of features at a global level ("$E_{globalC_{i}}$") is the optimal subset of features found globally across all clans or iterations, using scaling factors "*K*" that determines how strongly the elephant’s solution is influenced by the best feature subset within its clan and "*m*" that determines how much influence the globally best feature subset has on the elephant’s solution.


$$E_{new_{m}}=E_{current_{m}}+\emptyset *\left(E_{cenc_{i}}-E_{current_{m}}\right)$$ (2)

This Eq 2 represents how the fittest elephant, often referred to as the matriarch, $E_{new_{m}}$ is updated position of the matriarch based on the variance from its present location $E_{current_{m}}$ and the location of the central elephant $E_{cenC_{i}}$ in the clan. The update is scaled by the factor "∅," which determines the strength of the influence of the center elephant on the matriarch’s position.


$$E_{cenC_{i}}=\frac{1}{N}*\sum_{i=1}^{N}(E_{C_{i}})$$ (3)

This Eq 3 calculates the centroid feature *E*_*cenCi*_ subset by taking the average of all the feature subsets *N* within the clan "ci." and $\sum_{i=1}^{N}(E_{C_{i}})$ represents sums up each feature across all subsets in the clan and then divides by the number of subsets to obtain the centroid.

#### 3.4.2 Clan dispersal behavior

As male elephants reach a certain age, they tend to exhibit a behavior where they depart from the clan and venture out independently. This phenomenon can be simulated within the optimization algorithm by considering elephants with lower fitness values as representative of this behavior. In this way, the departure of male elephants from the clan can be emulated through the algorithmic processes, influencing how certain individuals interact and contribute to the optimization process.


$$E_{low_{m}}=\alpha *E_{cenC_{i}}+(1-\alpha )*E_{current_{m}}$$ (4)

In this Eq 4, the male elephants’ behaviour is implicitly represented by the balance between the centroid position and the current matriarch’s. $E_{low_{m}}$ Adjust the placement of the most robust elephant matriarch, *E*_*cenCi*_ is the centroid feature subset for the clan "*ci*", $E_{current_{m}}$ is the present position of the most robust elephant matriarch. The value of α can be adjusted between 0 and 1 to control the extent to which male elephants’ influence affects the matriarch’s position update. The centroid is given more weight when *α* is greater, whereas the matriarch’s current position is given more weight when *α* is lower.

Algorithm 1 provides the algorithm for the EHO based feature selection. Initialization of parameters including population size (N), maximal number of iterations (MaxIter), herd size (H), exploration rate (CR), and exploitation rate (MR) occurs at the outset of the algorithm. Subsequently, a population of feature subsets is generated at random to represent potential solutions. Each iteration involves elephants in the herd perturbing their current solutions in order to investigate the feature space, and subsequently assessing the fitness of the subsets that result. Optimal solutions are subsequently updated. Additionally, knowledge sharing among elephants is incorporated into the algorithm to increase search diversity. In conclusion, convergence is assessed and the optimal feature subset discovered is returned.

### Algorithm 1.Working principle of EHO technique

**INPUT:**
Size of the population = N
Maximum number of iteration = MaxIter
Herd size = H
Rate of Exploration = CR
Rate of Exploitation = MR
**PROCESS:**
**Initialize Population:**
Create N randomly selected feature subsets.
**Repeat for MaxIter iterations:**
For each elephant in the herd:
 **Explore:**
NewSubset = Perturb(CurrentSubset, CR)
**Exploit:**
Fitness = EvaluateFitness(NewSubset)
BestSubset = UpdateBestSolution(BestSubset, NewSubset)
 **Share knowledge:**
AdjustPosition(ElephantPosition, MR)
**Convergence Check:**
 If MaxIter iterations are reached, stop the optimization process.
**OUTPUT:**
Return the best feature subset found during the optimization process.

### 3.5 Proposed architecture and workflow

**Fig 3:**
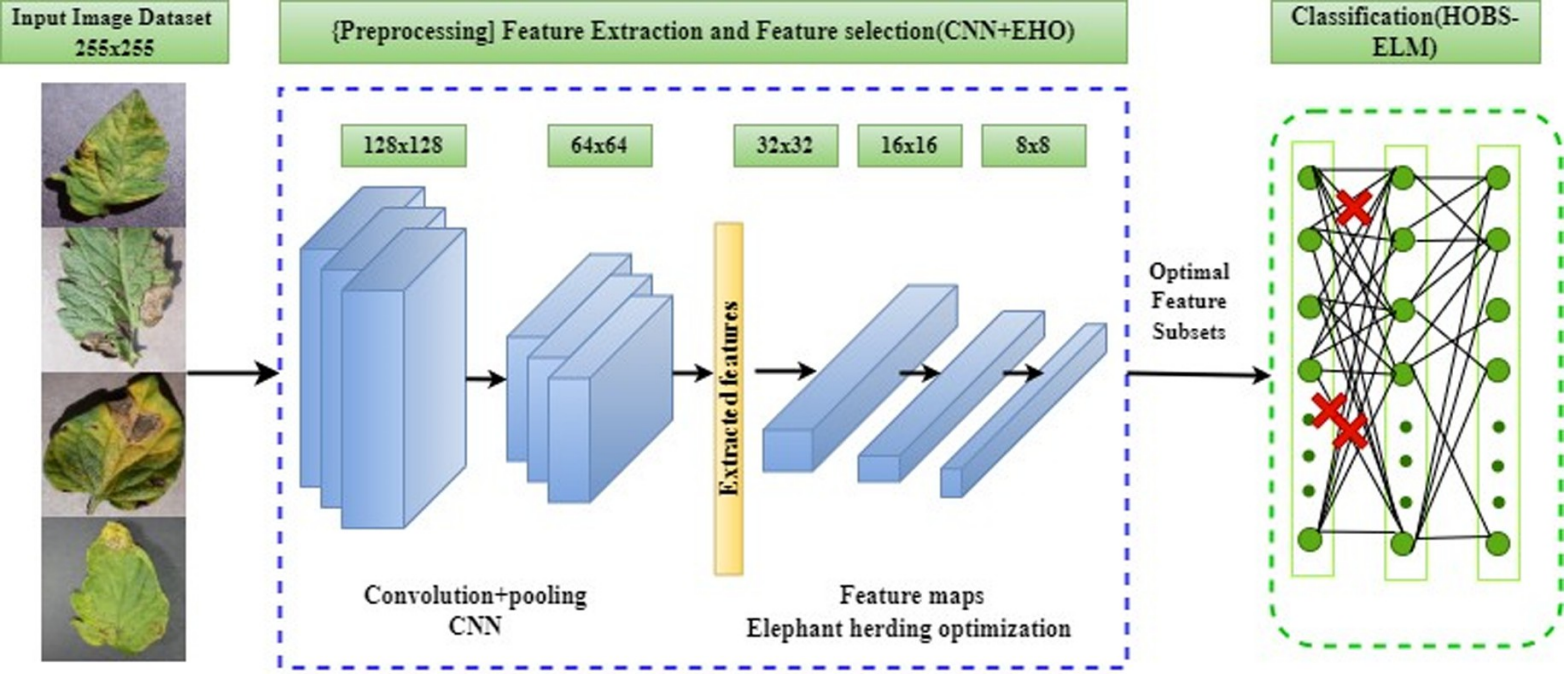
Proposed CNN (EHO+HOBS-ELM) architecture.

The initial stage of the process starts with gathering tomato leaf images, including both healthy and diseased samples. The subsequent phase encompasses preprocessing tasks applied to these input images. Moving to the third stage, the approach concentrates on isolating the diseased portion within an input image. After segmentation, the segmented portion of the diseased plant’s leaf image is used to compute and extract texture features, which highlight certain characteristics of the individual pixels or textures. The outlined method is visualized in Fig 3, through a block diagram.

The features extracted play a pivotal role in the subsequent analysis. Subsequently, with the goal of decreasing redundancy, a feature selection process takes place to choose the most relevant features that accurately reflect the provided image. After that, the type of disease is determined using these selected characteristics. Using a classification algorithm, the final step of this strategy is to identify the specific disease impacting the input plant leaf image [45]. Further elaboration on each of these phases of the proposed approach is provided below.

The decision to commence pruning from the final CNN layer is rooted in empirical evidence demonstrating that its neuron connections exhibit a notably lower degree of correlation within each class when compared to the other layers, evaluated by Pearson’s correlation coefficient, a statistical metric that quantifies the linear association between two continuous variables. Pearson’s correlation coefficient ranges from -1 to +1, with -1 signifying a perfect negative correlation, +1 denoting a perfect positive correlation, and 0 representing the absence of correlation. This last layer, essentially the classification layer, also coincides with the role of ELM in serving as a means for dimensionality reduction. This reduction operates along the neuron directions, enabling the pruning of connections at this level. Rather than employing a "masking out" approach for smaller weights, direct pruning at the neuron level proves to be more efficient, resulting in significant savings in both space and time.

#### 3.5.1 ELM basic principle

The single-layer feed forward neural network (SLFN), known as ELM [46], is highly regarded for its outstanding capabilities in regression and multi-class classification applications. ELM’s theoretical underpinnings underscore the notion that the hidden neurons can be generated randomly. In contrast to the SLFN, where both input and output layer weights are initialized randomly and subsequently adjusted through the back-propagation algorithm, ELM adopts a different approach. Through setup and training, the hidden layer weights in ELM are fixed to a random value. Unlike SLFN, where both layers undergo adjustments, ELM concentrates solely on updating the output layer weights. This distinctive feature, where only one layer’s weights are modified in ELM, gives it a significant speed and efficiency advantage [47].

In the scenarios of Extreme Learning Machines (ELM), the training dataset contains input vectors *x*_*j*_ and their corresponding output vectors *t*_*j*_. A group of neurons defines the network’s hidden layer. Each characterized by weight vectors *w*_*i*_, biases *b*_*i*_, and an activation function *g*. These neurons within the hidden layers are coupled to those in the final layer through connections by specific weights expressed in the symbol as *β*_*i*_.

The relationship among the hidden, output, and input layer can be stated as in Eq 5

$$\sum_{i=1}^{l}\beta_{i}g\left(w_{i},b_{i},x_{j}\right)=y_{j}$$ (5)

Where, *L* denotes the hidden neurons count, and j signifies the input/output instance, and *y*_*j*_ is the output corresponding to input *x*_*j*_. This relationship can be compactly represented as in Eq 6

Hβ=T (6)

Eq 7 represents the arrangement of matrices *β* and *T* in the context of the ELM model. Specifically, for *m* output nodes and *L* hidden neurons, the matrices *β* and *T* are structured as follows:

$$\beta ={\left[\begin{array}{c} \beta_{1}^{T}\\ \vdots \\ \beta_{1}^{T} \end{array}\right]}_{L\times m}\;and\;T={\left[\begin{array}{c} y_{1}^{T}\\ \vdots \\ y_{N}^{T} \end{array}\right]}_{N\times m}$$ (7)

Here, *β* is a matrix with the weights that link output neurons with hidden layer neurons, and *T* is the matrix containing all output values for the given inputs. The hidden layer’s output, as represented by Eq 8, is represented by the matrix H.


$$H=\left[\begin{array}{ccc} g\left(w_{1}\cdot x_{1}+b_{1}\right) & \cdots & g\left(w_{L}\cdot x_{1}+b_{L}\right)\\ \vdots & \ddots & \vdots \\ g\left(w_{1}\cdot x_{N}+b_{1}\right) & \cdots & g\left(w_{L}\cdot x_{N}+b_{L}\right) \end{array}\right]$$ (8)

With the use of the generalized inverse of Moore-Penrose matrix *H*, represented as *H*^+^ in Eq 9, one may determine the ideal output layer weights *β*.


$$\hat{\beta }=H^{+}T$$ (9)

The computation of *H*^+^ can be done through methods like QR decomposition, singular value decomposition (SVD), orthogonalization, or orthogonal projection, enabling efficient learning without the need for extensive computational resources. ELMs are advantageous due to their ability to perform well with a large number of hidden neurons, reducing computational complexity. Moreover, ELMs simplify the network architecture by eliminating the need for determining optimal hidden layers and bypassing the challenges associated with back propagation. However, a notable challenge in ELMs is identifying the least important connections within the network.

#### 3.5.2 HOBS- ELM pruning based classification process

**Fig 4:**
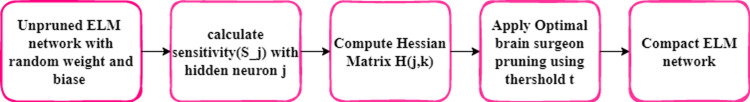
Working procedure of ELM classifier network.

The core concept behind HOBS-ELM lies in the assessment of the relevance degree among hidden nodes, subsequently eliminating connections deemed irrelevant or of low relevance. This leads to the creation of more compact networks, all while preserving the network’s generalization capabilities. In this architecture, the strategy consists of regularizing methods to find and remove less important connections between the ELM’s hidden layer and output layer. Afterward, a pruning method is implemented to enhance the network’s structure by refining the chosen connections. The Hessian-based Optimal Brain Surgeon (HOBS) [48] algorithm serves as the pruning tool for hidden connections. HOBS leverages the Hessian matrix to gauge the sensitivity of the network’s output concerning the hidden layer connections, thereby excising connections with lower sensitivity values. Every step in our proposed approach, as outlined in Algorithm 2, is non-iterative. This model offers a systematic and automated approach for precise classification while effectively addressing computational efficiency concerns. Furthermore, through experimentation the optimum amount of hidden layers and neurons is chosen to improve the model’s convergence speed. The learning process of this proposed method can be executed via the workflow procedure illustrated in Fig 4.

The first step is to start with an unpruned ELM neural network that links the hidden layer h to the input using random weights and biases (*W*,*B*). calculating the hidden layer *H* of output matrix requires knowledge of the input data matrix *X*, which has dimensions *N x D*, an activation function *g*, and *N* total amount of input feature mappings.

In step 2, analyze the sensitivity *Sj* for each hidden neuron *j*. For each input feature *i* calculate the absolute value of the weight connecting that feature to hidden neuron *j* is |*W*[*i*][*j*], Sum up these absolute weight values for all input features to get the total contribution of weights for neuron j is Σ*i*|*W*[*i*][*j*]|.

In the next step, perform pruning using the Hessian Matrix ***H*** (***j*,*k***) this matrix elements are computed. This represents an element in the Hessian matrix corresponding to the interaction between hidden neurons j and k. Here Σ*i* denotes the summation over all input samples(*i*). In the *i*_*th*_ input sample, *H*[*i*][*j*] represents the hidden layer activation value for hidden neuron *j*. In the input sample with index *i*_*th*_,*H*[*i*][*k*] represents the hidden layer activation value of hidden neuron *k*.

### Algorithm 2. HOBS- Pruned ELM

**Step 1: Unpruned ELM Network**
 Multiply input data matrix with weight matrix add bias then apply activation function g to find the resultant matrix.
 ***H* = *g*(*XW*+*B*)**
**Step 2: Calculate Sensitivity *S***_***j***_
Dividing its total absolute weight contribution by the sum of total absolute weight contributions for all hidden neurons ***S***_***j***_ = (**Σ**_***i***_**|*W*[*i*][*j*]/(Σ**_***k***_**Σ**_***i***_**|*W*[*i*][*k*])**
**Step 3: Compute Hessian Matrix H(j, k)**
The average of the pairwise products of elements from columns j and k in matrix H, with the sum taken over all rows, divided by the number of rows (N) ***H*(*j*,*k*) = Σ**_***i***_(*H*[*i*][*j*]*H[*i*][*k*])/*N*
**Step 4: Apply Hessian based Optimal Brain Surgeon Pruning**
Calculate the diagonal of the Hessian matrix D, sensitivity matrix S, saliency vector C, pruning threshold t is ***D* = [*H*(1,1),*H*(2,2),….,*H*(*h*,*h*)]**, ***S* = [*S***_**1**_**,*S***_**2**_**,…,*S***_***h***_], *C* = D*S, *t* = α*max(*C*).
**Step 5: Compact ELM Network**
If ***C*[*j*] < *t*** prune the neuron by excluding its associated weight and bias, or else keep the neuron and its associated weight and bias.

Applying the HOBS pruning step involves calculating the importance of connections using the Hessian matrix and sensitivity values. The saliency vector C captures the combined importance of each connection, and the pruning threshold t is determined as a percentage of the maximum saliency value, where α is a hyper parameter. This threshold helps identify connections to prune, and create a more compact neural network while retaining its important components.

The HOBS algorithm effectively identifies and prunes less important hidden connections, leading to a more compact and efficient neural network with potentially improved generalization capabilities. The specific choice of pruning threshold (t) and the number of retained connections may require experimentation and validation to achieve the best performance on the given dataset and task. The connection pruning method relies solely on straightforward matrix calculations to implement.

## 4. Experimental results and findings

The datasets collected were imported into the Google Colab environment for experimentation. For data analysis and model development, Numpy and TensorFlow were employed in conjunction with the Keras framework. The entire deep network was trained end-to-end on an NVIDIA 2070 GPU, utilizing the ADAM (Adaptive Moment) optimization algorithm and employing a batch size of 32. Table 2 presents the hyper-parameters employed in our study. The final model architecture consists of convolutional neural network (CNN) layers, each containing hidden states, along with a single dropout layer. The output layer was configured as the HOBS-ELM layer.

**Table 2:** Proposed model training parameters.

Network Hyper parameters	Values
Epoch	50
Learning rate	0.001
Batch size	32
Optimizer	ADAM
Activation function in hidden layer	ReLu
Activation function in output layer	Softmax

### 4.1 Hyper parameter tuning for proposed model

To improve classification accuracy, the proposed model’s hyper parameters are fine-tuned during training. These hyper parameters have a substantial impact on the training process, which improves the model’s capacity to learn and generalize properly [49].

**Learning rate:** If the learning rate is below the optimal level, the model will need additional epochs (hundreds or even thousands) to reach the optimal state. Conversely, if the learning rate is much higher than optimal, the outcomes may exceed the optimal state, potentially leading to calculation divergence.

**The number of epochs (training iterations):** Train the model for 50 epochs, allowing it to iteratively learn from the training data and adjust its internal parameters to minimize loss. This iterative procedure ensures that the model converges to an optimal state while increasing classification accuracy.

**Activation Functions:** The input layer utilizes the Rectified Linear Units (ReLU) activation function. The ReLU activation function introduces non-linearity to the model, enhancing its ability to properly capture intricate correlations within the data. This function optimizes the process of extracting features and improves the model’s capacity to comprehend a wide range of patterns within the input data. The output layer utilizes the softmax activation function. Softmax function is used to normalize the output scores for different classes, resulting in a probability distribution over the classes. This ensures that the model’s classifications are understandable and accurately reflect the probability of each class being the correct label.

**Selecting an Optimizer:** AdaM optimizer is used in the training phase. It enables effective convergence and quicker training by combining the benefits of momentum and RMSprop optimization approaches. Through adaptively changing learning rates for specific model parameters, AdaM facilitates the study of complex error surfaces and increases the process of convergence towards the optimal respond. It aim to maximize the performance of our suggested model by carefully adjusting these hyper parameters, ensuring accuracy, efficiency, and adaptability in the classification of diseases affecting tomato plants.

### 4.2 Performance metrics

The present study uses a benchmark dataset of images of tomato plant leaves to evaluate the efficiency of the proposed approach. In the split of the data set, training took up 80% of the images, while testing used up the remaining 20%. Within this dataset, the diseased plant leaf images exhibit afflictions stemming from one of ten distinct diseases. The performance metric quantifies the proximity between the original diseased plant leaf and its corresponding classified counterpart. Error rate, Kappa coefficient, training time, and testing time were regarded as additional evaluation measures for our suggested technique in addition to accuracy. When dealing with multiple classes, the accuracy metric computes the mean of individual class accuracies.


$$Accuracy=\frac{TPs+TNs}{TPs+FPs+TNs+FNs}$$ (10)

The error rate is the complement of accuracy, representing the proportion of misclassified instances. It provides an alternative view of the model’s performance.


$$Error\;rate=\frac{FPs+FNs}{TPs+FPs+TNs+FNs}$$ (11)

TPs (true positives) indicate the overall count of true instances accurately classified across all classes. TNs (true negatives) represents the total amount of instances that were accurately classified as not included within any category. FPs (false positives) stands for the total number of erroneously classified instances within a given category. FNs (false negatives) represent the total instances inaccurately classified as not belonging to any category.

Cohen’s Kappa is a statistical measure that evaluates the agreement between predicted and actual classifications while considering the possibility of chance agreement.


$$K=\frac{P_{o}-P_{e}}{1-P_{e}}$$ (12)

*p*_*o*_ (Observed Proportion of Agreement) is the relative observed agreement between the predicted and actual classifications. *P*_*e*_ (Expected Proportion of Agreement by Chance) is the proportion of agreement expected by random chance. These performance metrics offer a thorough assessment of the effectiveness, efficiency, and reliability of the EHO-based HOBS-ELM model in classifying diseases in tomato plants.

### 4.3 Correlation matrix for EHO feature selection

EHO stands as an optimization algorithm, drawing inspiration from the cooperative dynamics observed in the natural world among elephants. Regarding the particular topic of classifying tomato plant diseases, its leverage the EHO to pinpoint the dataset’s most pertinent attributes associated with tomato plants. Our approach involves the construction of a correlation matrix, facilitating an assessment of the interdependencies among various dataset features. The primary objective revolves around the identification of features that exhibit substantial correlations with distinct disease categories, such as bacterial spots, late blight, and early blight. The rationale here is to unearth those features that promise valuable information for the classification task.

*C*1 and *C*2 are two correlation variables that can be calculated using Pearson’s correlation coefficient, which is a common method for measuring linear relationships as shown in Eq 13.


$$(\rho )=\sum \left(\left(C1-\mu_{C1}\right)\left(C2-\mu_{C2}\right)\right)/\left(\sigma_{C1}*\sigma_{C2}\right)$$ (13)

Where *C*1, *C*2 are the variables being correlated. *μ*_*c*1_,*μ*_*c*2_ are the ‘means’ of *C*1 and *C*2 respectively. *σ*_*c*1_,*σ*_*c*2_ are the standard deviations of *C*1 and *C*2 respectively.

**Fig 5:**
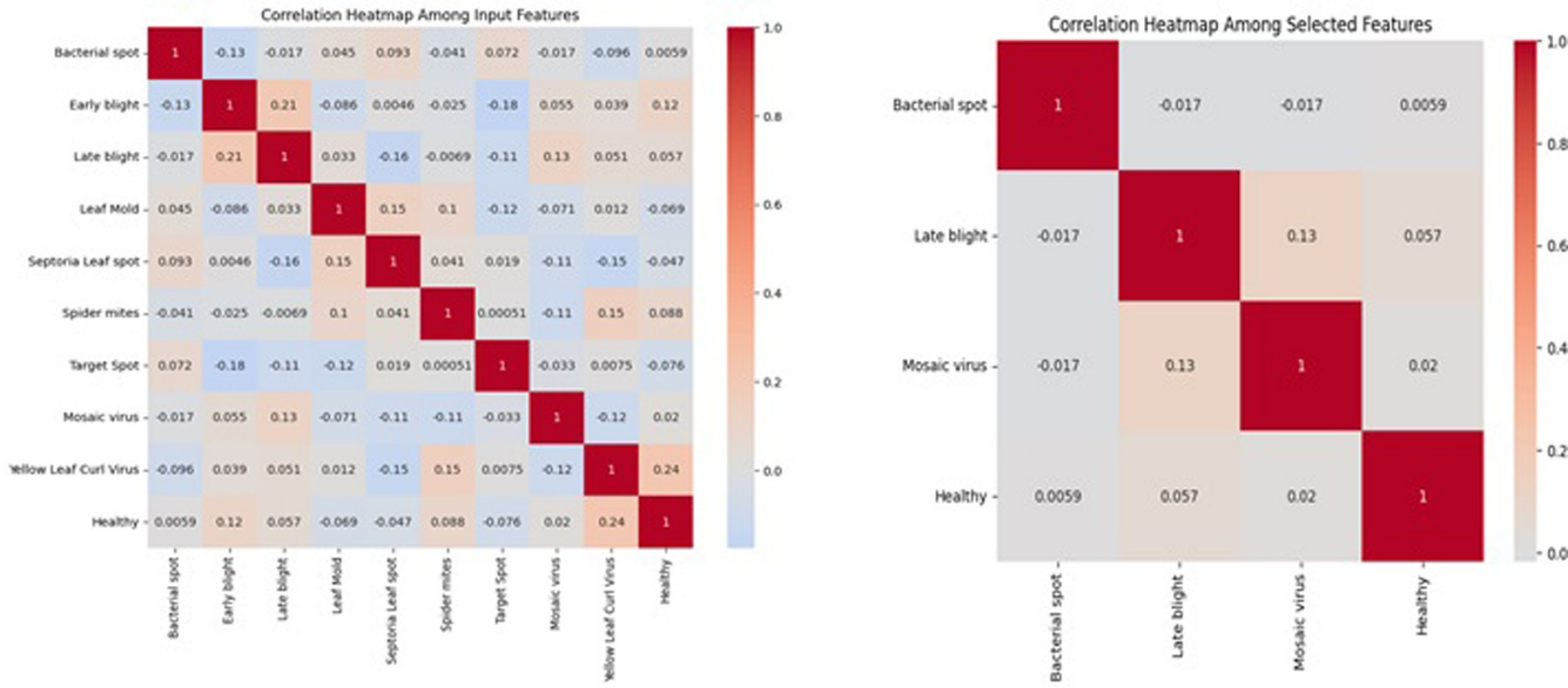
Correlation heat map for feature selection.

Fig 5, visually depict the correlation matrix, generate a heat map where each cell signifies the correlation between two distinct features. Elevated positive correlations are typically depicted using warmer shades, notably the colour red, while strong negative correlations are represented by cooler hues, such as blue. This heat map presents a visual synopsis of the interrelationships among various attributes, shedding light on their potential influence on the classification process.

The correlation matrix and heat map serve as vital tools in comprehending the intricate interplay among attributes and their impact on precise disease categorization. These tools assist in the identification of attributes that not only provide information but also take into account their mutual interactions and dependencies. Through the visualization of correlations, valuable insights emerge regarding which characteristics wield the most influence in distinguishing among distinct disease categories, thereby facilitating feature selection. The attributes chosen based on their correlations with disease classes can then be harnessed to construct a more efficient model for categorizing tomato plant diseases. This process deepens our comprehension of attribute interdependencies and their consequential importance, ultimately guiding informed decision-making during the feature selection phase.

The correlation heat map provides critical insights into the relationships between the selected features. Bacterial spot exhibits minimal correlation with other diseases, indicating that it can be classified as a distinct class. The characteristics of late blight and Mosaic virus exhibit a moderate positive correlation, suggesting that there may be some overlap. This could potentially impact the performance of classification. The correlation analysis shows that numerous features exhibit minimal correlations with one another. This implies that the selected features make a distinctive contribution to the classification of diseases. This is expected to lead to improved classification accuracy in the model, as a result of the reduced redundancy and the more distinct distinctions between disease categories.

### 4.4 Classification analysis

A series of experiments were carried out using three different algorithms to assess the performance of our proposed approach. Deep convolutional extreme learning machine (DC-ELM), CNN, and HOBS-ELM. These experiments were performed on the widely used Plant Village dataset, a benchmark collection in the realm of agricultural image categorization. The dataset is divided into ten categories, each of which corresponds to a distinct tomato plant disease. Table 3 showcases the results of classification accuracy obtained from different network models when applied to individual tomato plant diseases within the Plant Village dataset. The experimental results demonstrate that the proposed model, which includes feature selection, consistently outperforms both the CNN and Deep Convolutional ELM models across a variety of tomato plant disease categories. This significant improvement in classification accuracy, as demonstrated in each disease class, underscores the efficacy of the proposed approach in enhancing model performance for precise disease classification.

**Table 3:** Classification accuracy results for each class in the tomato plant disease dataset.

Class	No of training samples	No of testing samples	CNN (pre-trained)	Deep Convolutional -ELM	With feature selection (EHO+HOBS-ELM)
Bacterial spot	800	200	78.89	74.88	88.23
early blight	800	200	69.06	93.86	100.00
Late blight	800	200	83.99	73.27	99.24
Leaf Mold	800	200	90.38	77.50	95.84
Septoria leaf spot	800	200	80.17	92.86	87.14
Spider mites	800	200	79.25	87.45	97.31
Target Spot	800	200	73.51	87.03	89.06
mosaic virus	800	200	66.77	90.43	96.83
Yellow Leaf Curl Virus	800	200	54.96	87.96	98.88
Healthy	800	200	86.16	91.50	97.78

Table 4, illustrates the outcomes of various network models applied to tomato plant leaf disease classification after retraining our model following HOBS pruning. The results clearly demonstrate the significant impact of model compression on reducing the model’s dimensions and computational burden while simultaneously elevating accuracy. In specific application scenarios, speed is a crucial performance metric. The pruning algorithm effectively compresses the model based on the designated pruning ratio, streamlining the network for optimal efficiency.

**Table 4:** Performance analysis of various model on the tomato plant dataset.

Algorithm	Over all Accuracy	Cohen’s Kappa	GFLOPs	Parameter	Model size (MB)	Training Time(s)
CNN	75.31	0.63	3.212	18.560M	73.3	35.92
DC-ELM	86.67	0.80	1.892	8.120M	33.5	28.19
(EHO+HOBS-ELM)	95.73	0.81	1.742	7.955M	9.2	2.35

The connection between accuracy and the pruning rate within the framework of tomato plant disease classification using the HOBS-ELM is a crucial aspect to highlight because two pruned networks undergo a single retraining iteration, ensuring an equitable comparison. Furthermore, to maintain parity in this evaluation, the pruned ELM (Extreme Learning Machine) layers are deployed for the purpose of classifying the optimized feature subset. The main objective is to simplify the classification layer by preferring alternative, lighter classifiers. It’s important to clarify that the reported percentage of pruning exclusively pertains to the ELM layers. To guarantee consistency, all shared hyper parameters are configured identically for both approaches, with occasional fine-tuning of parameters introduced only when necessitated. It’s worth noting that periodic adjustments to the batch size may be warranted to steer clear of local minima.

**Fig 6:**
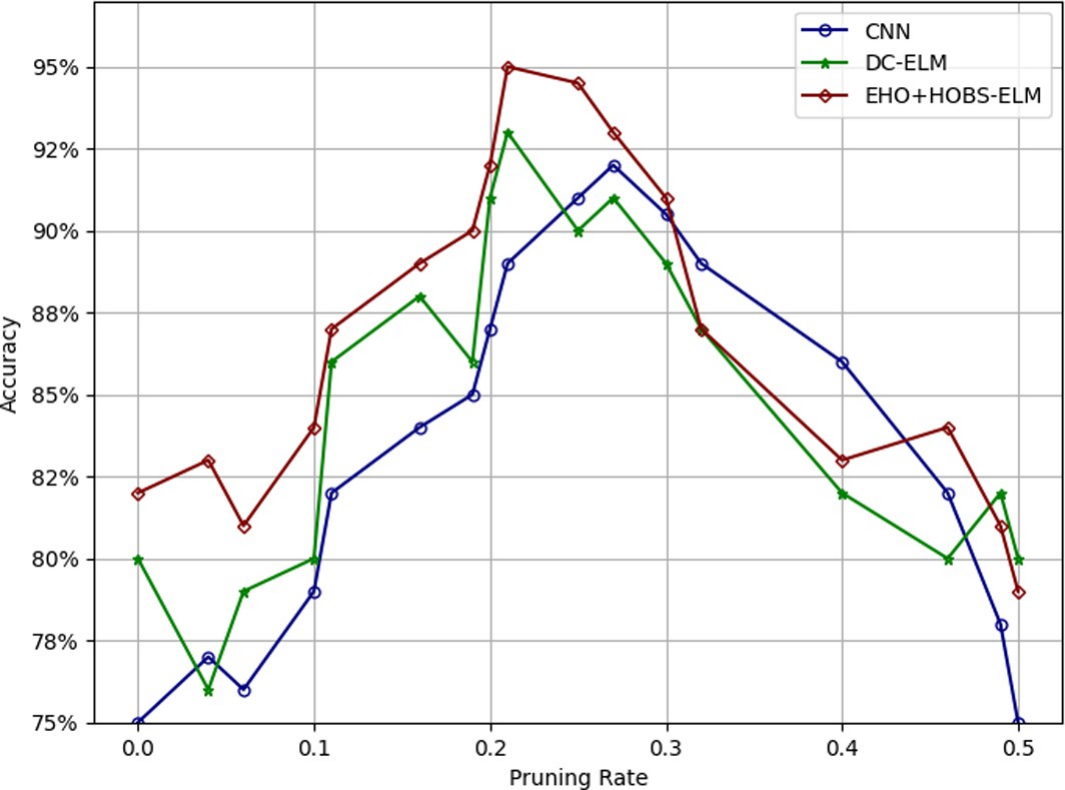
Accuracy change vs. ELM layers pruning rate.

As depicted in Fig 6, the approach consistently outperforms the alternative method across various pruning rates. Notably, in some instances, this approach even exhibits a marginal accuracy enhancement. This superior performance can be ascribed to the method’s inherent understanding of each neuron’s contribution to the ultimate discriminative power during the pruning process, taking into account interdependencies spanning all layers. In contrast, the alternative method relies solely on the length of a single layer’s dependencies, potentially leading to the exclusion of diminutive weights that, in reality, contribute to more informative neurons in the final layer. This arises because minor weights may possess cumulative effects across multiple layers or might be amplified by larger weights in other layers. Pruning these minor weights within a single layer effectively severs entire connections, starting from the raw pixel level and extending to the ultimate classification stage. While weight magnitude indeed serves as a valid pruning criterion, it should not serve as the sole determinant when evaluating the significance of a comprehensive bottom-to-top connection, especially within the context of HOBS-ELM; the result serves to classify tomato plant leaf diseases. While the classification module significantly enhances the model’s classification accuracy, it does so without introducing additional parameters and complexity. However, it is essential to understand that the existing parameters and complexity of the model can still pose challenges for deployment on edge devices with constrained computational resources. To address this issue and decrease the model’s parameters and complexity, the proposed HOBS pruning on the model specifically targets the enhanced ELM module.

**Fig 7:**
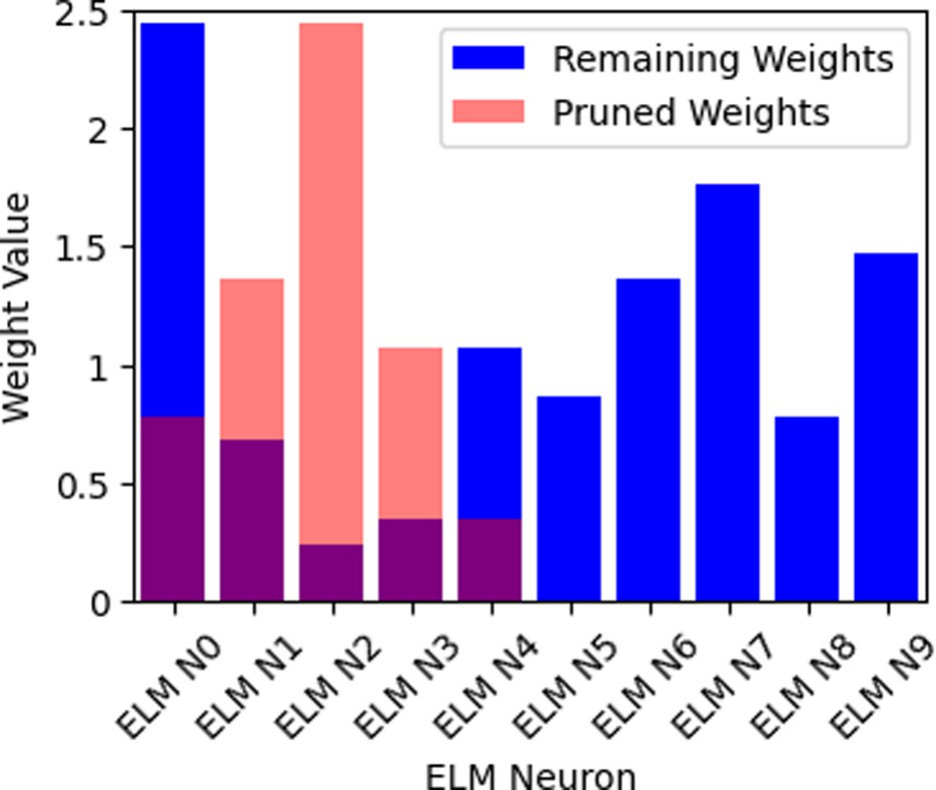
Layer wise structure complexity reduction.

Table 5 presents the outcomes obtained through retraining the model following various levels of pruning. Model compression offers substantial reductions in model size and computational demands while simultaneously showcasing noteworthy improvements in accuracy. To gain a deeper understanding of our pruning technique’s nuances, Fig 7, and Table 5 furnish a comprehensive layer-wise examination of the speedup ratio. Fig 7, visually underscores that the bulk of parameters in the final ELM layers (ELM N5 to ELM N9) do not exert a significant influence on our designated task. Conversely, in contrast to the preceding layers, the last four layers exhibit relatively modest reduction rates. This observation aligns with the prevailing knowledge that earlier layers tend to encompass most common features like edge and color blob detectors, which hold the potential for utility across all classification categories.

**Table 5:** Comparative analysis of layer, parameters and speedup ratio for original CNN+FC and proposed HOBS-ELM models.

Method Layer	Original CNN+FC	Proposed HOBS-ELM	Speedup Ratio
Convolutional Layer	1000 (e.g., Conv1, Conv2, etc.)	1000 (e.g., Conv1, Conv2, etc.)	1.00
Fully Connected Layer 1	1000 (4096 neurons)	150 (HOBS-ELM Layer)	6.67
Fully Connected Layer 2	800 (4096 neurons)	150 (HOBS-ELM Layer)	5.33
Final Output Layer	100 (100 neurons)	150 (HOBS-ELM Layer)	0.67
Overall	Total: 2900	Total: 1450	2.00

Plant leaf disease classification methods are compared by computing efficiency and accuracy in the Table 6. The suggested method achieves 95.73% classification accuracy with little computing load. Adaptive feature selection with deep learning MBi-LSTM has reasonable computing efficiency and 94.20% accuracy. Efficient adaptive feature selection with deep learning model has low processing requirements and 94.80% accuracy. Finally, the Hybrid optimization method using RBFNN and ABC has modest computing efficiency and 92.50% accuracy. The hybrid approach presents a promising compromise between computing efficiency and classification accuracy compared to existing feature selection and optimization strategies.

**Table 6:** Feature selection and pruning strategies comparison.

References	Technique	Computational efficiency	Classification accuracy
[50]	Adaptive feature selection with deep learning MBi-LSTM model based paddy plant leaf disease classification	Moderate	94.20
[51]	Efficient adaptive feature selection with deep learning model-based paddy plant leaf disease classification	High	94.80
[52]	Automatic feature extraction process with machine learning based tomato plant leaf disease classification	Moderate	92.30
-	**Proposed (EHO+HOBS)**	Low	95.73

#### 4.4.1 Statistical analysis

Overall accuracy is the proportion of examples each algorithm successfully classifies across all classes. To account for chance, Cohen’s Kappa coefficient compares predicted and actual classifications. In imbalanced datasets or where accuracy alone may be misleading, this gives a robust classification performance measure. The amount of floating-point operations needed to process one second of data is used to measure the model’s computational complexity during inference. Real-time or resource-constrained applications benefit from lower GFLOPs indicating computational efficiency. Fewer parameters reduce over fitting and computational load by showing the model’s complexity and ability to learn from training data. Compact models are better for resource-constrained devices and efficient storage since they use less memory to hold model parameters. Computing efficiency and scalability are shown by model training time on the dataset. Large datasets and iterative model development make shorter training times preferable.

Table 7, shows the performance, computational complexity, and efficiency of the CNN, DC-ELM, and (EHO+HOBS-ELM) algorithms are assessed through statistical analysis using these metrics. The evaluation involves identifying the most appropriate algorithm for specific application requirements by comparing the trade-offs between accuracy, model complexity, and training/inference efficiency.

**Table 7:** Statistical summary of models performance metrics.

Parameter	Mean	Standard Deviation	Correlation with accuracy
**Overall Accuracy**	85.57	9.63	-
**Cohen’s Kappa**	0.74	0.09	0.89 (positive correlation)
**GFLOPs**	2.282	0.76	0.67 (positive correlation)
**Parameter**	11.878M	5.319M	0.73 (positive correlation)
**Model size (MB)**	38.66	30.34	0.58 (positive correlation)
**Training Time(s)**	22.81	15.97	-0.92 (negative correlation)

## 5. Discussion

To effectively apply convolutional neural networks to real-world agricultural production settings, it’s imperative that these networks operate in real-time, even on platforms with constrained processing power, limited memory, intricate architectures featuring various constraints, and filtering redundant feature maps. To overcome these obstacles, pruning is carried out on the CNN architecture’s classification module while preserving the HOBS-ELM block. Existing literature offers several approaches to eliminating less critical connections. However, many of these pruning techniques are better suited for straightforward network architectures. The pruned network then undergoes a retraining process, with all hyper parameters aligned with those employed in our previous experiments for plant disease classification. This methodology ensures the adaptability and efficiency of CNNs in the context of agricultural production and specifically in tomato cultivation without necessitating substantial architectural alterations.

A thorough performance comparison was made between the proposed approach and a recently published model, as shown in Table 8. This comparative analysis was grounded in metrics encompassing accuracy, FLOPs (floating-point operations per second), and model parameters. FLOPs act as a measure to quantify a model’s computational complexity. On the other hand, the model’s parameters are crucial in determining its physical size and memory requirements. Notably, the pruned network demonstrated a remarkable level of computational efficiency, as evidenced by FLOPs measurement, training speed, and inference time.

**Table 8:** Performance evaluation of the proposed technique with existing models.

References	Model	Accuracy of plant village	GFLOPs	Parameter	Model size(MB)
[7]	Pruned DenseNet	86.64	21.00	11.2M	10.6
[53]	Lightweight channel pruned CNN(GhostNet)	92.15	2.54	10.6M	10.9
[25]	CNN Channel attention and channel pruning(CACPNET)	92.4	1.819	11.18M	18.0
[36]	Swin transformer	99.4	-	86.8M	438
[36]	ResNet	96.18	-	23,5M	273
[36]	EfficientNet	94.73	-	28.4M	211
Proposed model	CNN(EHO+HOBS-ELM)	95.73	1.742	7.95M	9.2

**Fig 8:**
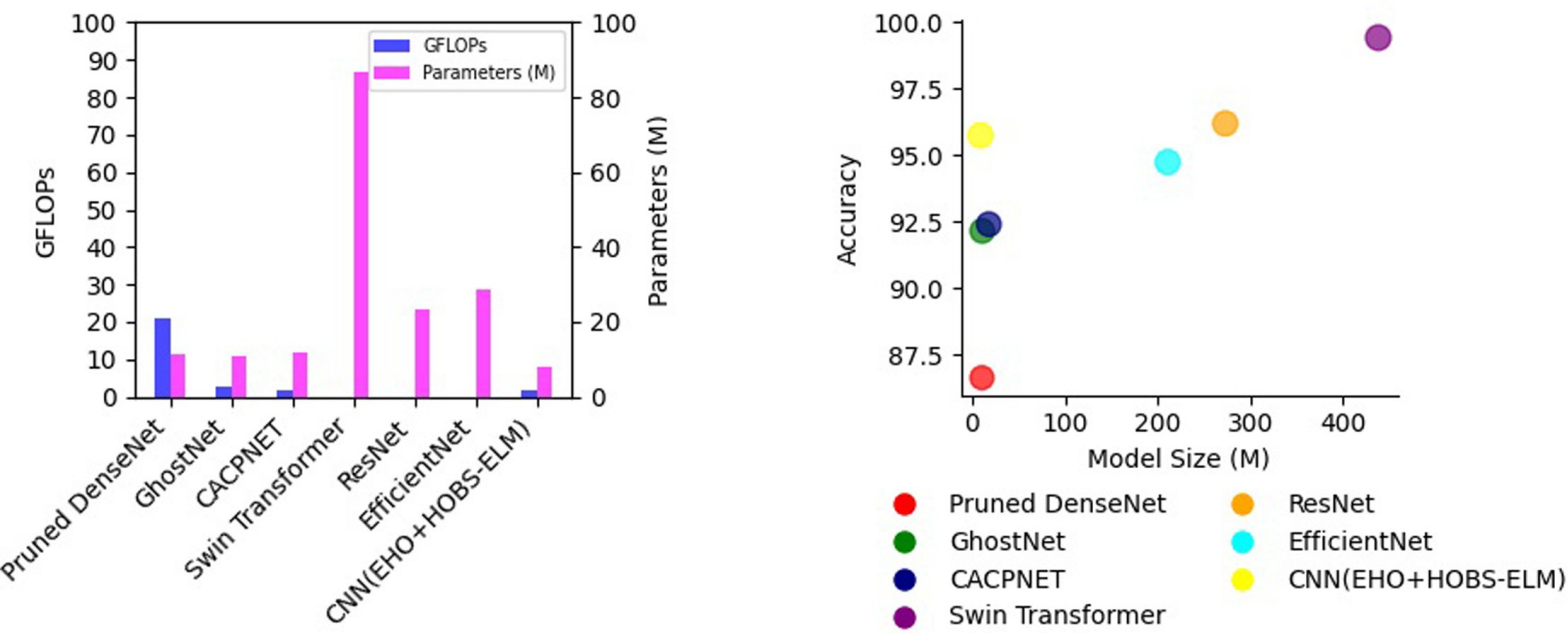
Model performance index analysis (a) Four models’ FLOP and parameter histograms (b) Four models’ model size and accuracy bubble charts.

The connection pruning algorithm inherent to the work excels in model compression, effectively reducing the count of parameters and FLOPs while maintaining a favorable pruning ratio (as illustrated in Table 4 and Fig 8(A). Consistency in training parameters was maintained to ensure a balanced and unbiased assessment of performance among models. This required using a weight decay of 0.001, using the SGD optimizer, setting the learning rate to 5e-4, and using CrossEntropyLoss as the loss function.

The performance of the (EHO+HOBS-ELM) model was tested with three learning rates: 0.1, 0.01, and 0.001. The model’s accuracy and Cohen’s Kappa coefficient vary significantly with learning rates. At 0.1 learning rate, the model had 94.50% accuracy and 0.79 Cohen’s Kappa, suggesting robust performance. Reducing the learning rate to 0.01 increased accuracy to 95.25% and Cohen’s Kappa coefficient to 0.80. The model performed best with a learning rate of 0.001, with an overall accuracy of 95.73% and a Cohen’s Kappa coefficient of 0.81. The impact of different data split ratios on the performance of the proposed algorithm was evaluated across various configurations.

The algorithm obtained 95.73% accuracy and 0.81 Cohen’s Kappa with an 80–20 data split ratio for training and testing. Using a 70–30 split reduced accuracy to 95.65% with same computing complexity. When using 60–40 split reduced accuracy to 95.50% while preserving computational needs. Allocating 80–20 data for training boosted accuracy to 95.73%. Optimizing data split ratios is crucial to algorithm performance due to the algorithm’s sensitivity to training and testing data distribution. Remarkably, even after pruning, the proposed model substantially outperforms other models, with significantly lower FLOPs and parameters. This noteworthy achievement highlights the ability to maintain high classification accuracy while achieving significant reductions in both model complexity and computational demands through connection pruning. Fig 8(B) displays the accuracy and model size requisites for all models under consideration. Notably, among these models, CNN (EHO+HOBS-ELM) exhibits the most minimal model size requirements. When compared to the other models, our proposed model stands out by showcasing reductions in FLOPs, parameters, and overall model size.

### 5.1 Comparative analysis of proposed work with previous literature

Additionally, an experiment was conducted to assess the model’s performance in classifying diseases from tomato leaf images. Table 9 shows the comparison with other state-of-the-art DL-based approaches. However, it’s worth noting that this comparison may not be entirely direct due to differences in training and testing methodologies, computational resources, and data preprocessing techniques employed in the respective methods.

**Table 9:** Comparative analysis of proposed approach versus baseline models.

Author	Methods	Accuracy (%)	Precision (%)	Recall (%)	F1-Score (%)
[46]	ELM	94	95	92	93.5
[27]	SVM	92	91	93	92
[35]	CNN-RNN	81.75	80	85	82.5
[28]	CNN	91.20	92	90	91
[29]	MobileNet v2-YOLOv3	92.53	91	94	92.5
[32]	ResNet101	95.83	96	95	95.5
[34]	CapsNet	94.47	94	95	94.5

To assess the generalizability and robustness of our proposed model, we conducted ablation studies on two additional datasets: rice leaf disease and bean leaf disease. Employing the same model architecture (CNN with EHO+HOBS-ELM), we observed accuracy scores of 94.20% for the rice leaf disease dataset and 93.50% for the bean leaf disease dataset. These results demonstrate the efficacy of our proposed model across diverse plant disease datasets, showcasing its potential applicability beyond the specific context of tomato plant leaf disease. Performing cross-dataset experiments further validates the robustness and effectiveness of our model in plant disease classification tasks.

### 5.2 Limitation and future work

There are notable limitations associated with this retrospective, unifocal investigation that needs to be addressed. The dataset used here is a subset of the Plant Village database, focusing solely on tomato plant diseases, which might not cover the full spectrum of plant diseases. Consequently, the findings and model performance may have limited applicability beyond tomato plant disease classification. To improve generalizability, future studies will include a larger and more diverse dataset covering a wider range of plant diseases. Additionally, efforts will be made to develop a more robust system suitable for real-world scenarios, as the current reliance on laboratory-generated images poses a significant limitation. Future research will also focus on refining the disease classification process for various plant diseases and automating the recognition of different disease stages, potentially incorporating images from natural environments. Additionally, future endeavors will involve augmenting the dataset of plant leaf images and implementing alternative validation techniques like K-fold cross-validation to enhance accuracy.

## 6. Conclusion

This investigation focuses on the automatic categorization of tomato leaf diseases using a pruned CNN model developed through combining an optimization technique with a pruned ELM model.

The primary objective was to overcome the complexity and accuracy limitations seen in existing models by providing a more efficient and accurate approach to tomato leaf disease classification. The proposed CNN (EHO+HOBS-ELM) model builds on the strengths of Extreme Learning Machines (ELMs) while using pruning strategies to minimize unimportant network connections. This pruning procedure minimizes the amount of the model’s parameters while increasing computational efficiency, ensuring that the network remains lightweight without sacrificing performance. The strategy improves the model’s parameter efficiency, making it more suitable for real-world applications.

The experimental results demonstrate that the model’s efficiency, with a classification accuracy of 95.73%. In addition to accuracy, the model addresses the significant issues of enormous model sizes and prolonged inference times, which are prevalent in deep learning models employed for this objective. The proposed approach demonstrates enhanced accuracy, faster inference times, and reduced parameter needs, establishing it as an effective and robust solution for the classification of tomato leaf diseases.

## Supporting information

S1 File (DOCX)
