# Supplementary material for: Effective feature selection based HOBS pruned- ELM model for tomato plant leaf disease classification
Source: PLoS One. 2024 Dec 5;19(12):e0315031. doi: 10.1371/journal.pone.0315031 (PMC11620619; doi:10.1371/journal.pone.0315031)
Supplement: S1 File — (DOCX) [file pone.0315031.s001.docx]

**Supporting information regarding Dataset**

**Title of Dataset:** Tomato leaf disease dataset.

**Dataset Link**

**GitHub link:** <https://github.com/MAmudha/Tomato-leaf-disease-dataset.git>

**Kaggle dataset link:** <https://www.kaggle.com/datasets/emmarex/plantdisease>

**Description:** The provided GitHub link leads to a collection of minimal images illustrating tomato plant leaf diseases, with each folder comprising set of images for reference. The images are useful resources for agricultural researchers and practitioners, allowing people to investigate and analyze various diseases that impact tomato plants.

**File format:** The format of the dataset file is image files.
